# Spontaneous Reorganization of DNA-Based Polymers in Higher Ordered Structures Fueled by RNA

**DOI:** 10.1021/jacs.1c09503

**Published:** 2021-11-29

**Authors:** Serena Gentile, Erica Del Grosso, Passa E. Pungchai, Elisa Franco, Leonard J. Prins, Francesco Ricci

**Affiliations:** † Department of Chemistry, University of Rome Tor Vergata, Via della Ricerca Scientifica, 00133 Rome, Italy; ‡ Department of Bioengineering, University of California at Los Angeles, 410 Westwood Plaza, Los Angeles, California 90095, United States; § Department of Mechanical and Aerospace Engineering and of Bioengineering, University of California at Los Angeles, 420 Westwood Plaza, Los Angeles, California 90095, United States; ∥ Department of Chemical Sciences, University of Padua, Via Marzolo 1, 35131 Padua, Italy

## Abstract

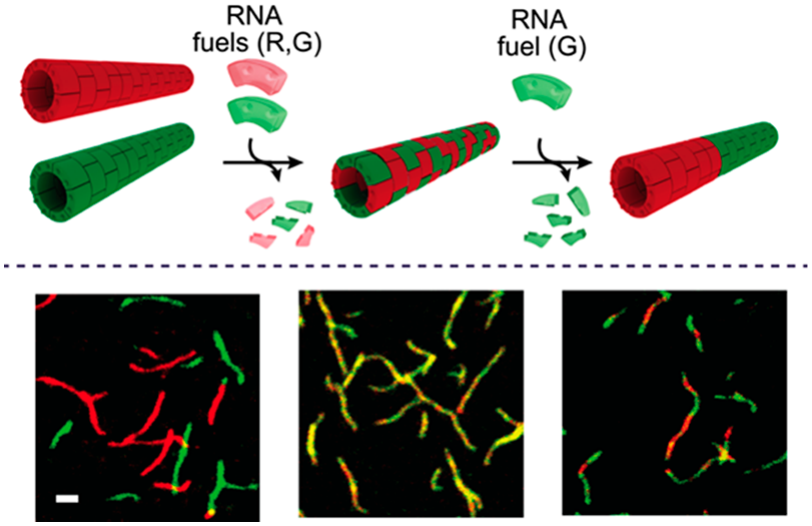

We demonstrate a strategy that allows for the spontaneous reconfiguration of self-assembled DNA polymers exploiting RNA as chemical fuel. To do this, we have rationally designed orthogonally addressable DNA building blocks that can be transiently deactivated by RNA fuels and subtracted temporarily from participation in the self-assembly process. Through a fine modulation of the rate at which the building blocks are reactivated we can carefully control the final composition of the polymer and convert a disordered polymer in a higher order polymer, which is disfavored from a thermodynamic point of view. We measure the dynamic reconfiguration via fluorescent signals and confocal microscopy, and we derive a kinetic model that captures the experimental results. Our approach suggests a novel route toward the development of biomolecular materials in which engineered chemical reactions support the autonomous spatial reorganization of multiple components.

## Introduction

Life is a nonequilibrium state of matter that is maintained at the expense of energy.^1^ Energy consumption allows the operation of molecular pumps^2,3^ and motors^4^ and drives the formation of self-assembled high-energy structures.^5,6^ A common fundamental element of these living biomolecular systems is that their operation is governed by kinetics rather than thermodynamics.^7,8^ This aspect has recently spurred the development of chemical systems that are likewise controlled by kinetic processes^9−15^ as an important stepping stone toward the development of synthetic materials with life-like properties.^16,17^ This trend is particularly evident in the synthesis of biomaterials designed to self-assemble from nanometer sized building blocks. Whereas traditionally the focus has been on the assembly product with the highest thermodynamic stability,^18^ recently it has been shown that different kinetic products can be selectively obtained from the same building blocks by carefully designing the supramolecular kinetic pathways leading to their formation.^19,20^ However, in these systems transitions between different kinetic states can occur only if these are energetically downhill, and furthermore, the systems are destined to eventually progress toward the thermodynamically most stable state after which they cannot be reactivated. These limitations can be overcome by using chemical fuels to kinetically control the self-assembly processes: specifically, building blocks for self-assembly are activated or deactivated by reactions requiring chemical fuel.^21,22^ This means introducing chemically distinct, transient pathways to regulate the activity of building blocks via batchwise addition of fuel.

We and others have recently shown that DNA offers tremendous opportunities in the field of chemically fueled self-assembly^23−28^ because (1) hybridization and strand exchange reactions are highly predictable from both a thermodynamic and kinetic point of view, (2) a myriad of enzymes are available to regulate nucleic acid fuel-to-waste conversion with high efficiency and selectivity, and (3) the multivalent nature of DNA hybridization generates a high tolerance to waste accumulation. These properties have been used to establish methods to transiently assemble and disassemble polymeric DNA structures, for example, as a result of enzymatic RNA production to activate components and promote their assembly and RNA degradation to cause the assemblies to collapse.^25−27,39^ Similarly, spontaneous DNA polymer assembly and disassembly can be achieved by using DNA nicking and ligation reactions.^29^

Another major advantage of DNA is that careful sequence design makes it possible to program distinct pathways that may be functionally or structurally similar but are individually addressable and operate orthogonally. This feature allows for the assembly of diverse components each responding to specific inputs or reactions; for example, different populations of DNA polymeric structures can be built, broken up, and reorganized by specific inputs that are provided sequentially to carry out these steps.^26,35^ The introduction of chemically fueled reactions in these multicomponent systems could support the synthesis of supramolecular materials that spontaneously control their organization.

Motivated by the above considerations, here we engineer a DNA polymeric system consisting of multiple, distinct assembling units (tiles), each of which controlled by distinct RNA fuels. The system takes advantage of the kinetics of enzymatic degradation of these RNA fuel species to determine the supramolecular organization of the units and induce a transition from disordered polymers into higher order polymers. This transition is autonomous and reversible. Our strategy allows us to shuttle the system in a controlled fashion between different kinetic states just by adding the appropriate RNA fuel and it permits the population of an energetically uphill state, which is entropically disfavored.

## Results and Discussion

**Figure 1:**
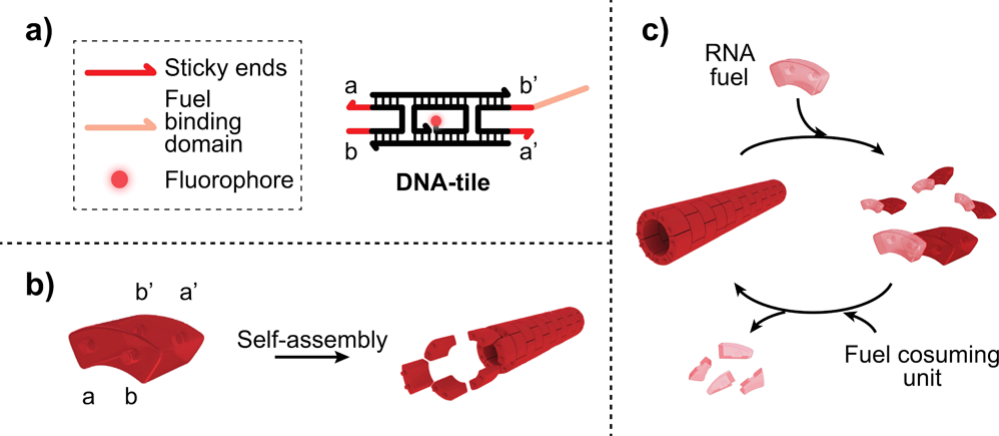
Spontaneous self-assembly and disassembly of DNA-based polymers triggered by RNA fuels. (a) DNA tile containing 5-nt sticky ends (red domains) and a fuel binding domain (light red domain). (b) DNA tile is pictured as a LEGO-like brick (knobs and holes represent the four sticky ends). The DNA tile self-assembles at room temperature into hollow tubular polymeric structures.^32,33,26^ (c) The RNA fuel (light red) invades the tiles leading to the disassembly of the polymeric structures. A fuel-consuming unit (RNase H) selectively degrades the RNA fuel when bound to the DNA tile inducing tile reactivation over time and reassembly of the DNA-based polymer.

To demonstrate the RNA-fueled reorganization of DNA-based polymers, we adopted the well-characterized double-crossover DNA tile, known as DAE-E.^30−32^ The tiles are formed through the interaction of five unique DNA strands and contain four sticky ends (5-nt each) (Figure 1a) that enable their self-assembly into tubular structures with an average diameter of 13.5 nm, 6–8 tiles along the circumference, and a persistence length of 4 μm (Figure 1b).^32,33,26^ The DNA tiles also contain a 7-nt binding domain that acts as an anchoring site for the binding of a 14-nt RNA strand (here termed fuel) capable of invading one sticky end portion and induce the complete disassembly of the tubular structure (Figure 1a–c and Figure S1).^26,34^ When the RNA fuel is bound to the complementary domain on the DNA tile, this RNA/DNA heteroduplex can be specifically recognized by the enzyme RNase H, an endoribonuclease enzyme able to selectively hydrolyze the RNA strand in the heteroduplex (Figure 1c and Figure S1). This enzymatic degradation of the RNA fuel reactivates the DNA tiles, which can reassemble into a new tubular structure (Figure 1 and Figure S1).

For this study, we developed a set of two orthogonally addressable tiles (red, R, and green, G) that have the same sticky ends but differ in the fuel-binding domain,^35^ so that each tile type can be recognized by a specific RNA fuel (red, R, and green, G). To independently monitor the assembly of each tile type, we labeled one of their strands with different fluorescent dyes (Quasar570 for the red tiles and Quasar670 for the green tiles) (Figure 1a). The combined use of both tiles for self-assembly can lead to different polymers depending on the organization of the tiles: homopolymers are structures that consist of one type of tile, block copolymers contain segments of tiles of the same type, and in random copolymers the two tiles are randomly distributed. Although the identical sticky ends in both tiles ensure that the enthalpy of formation is the same for all structures, from an entropic point of view the formation of ordered structures (homopolymers and block copolymers) is disfavored compared to the disordered random copolymer. Therefore, the homopolymers and block copolymers can be considered as kinetic products of the self-assembly process as opposed to the random copolymer, which is the thermodynamic product.

**Figure 2:**
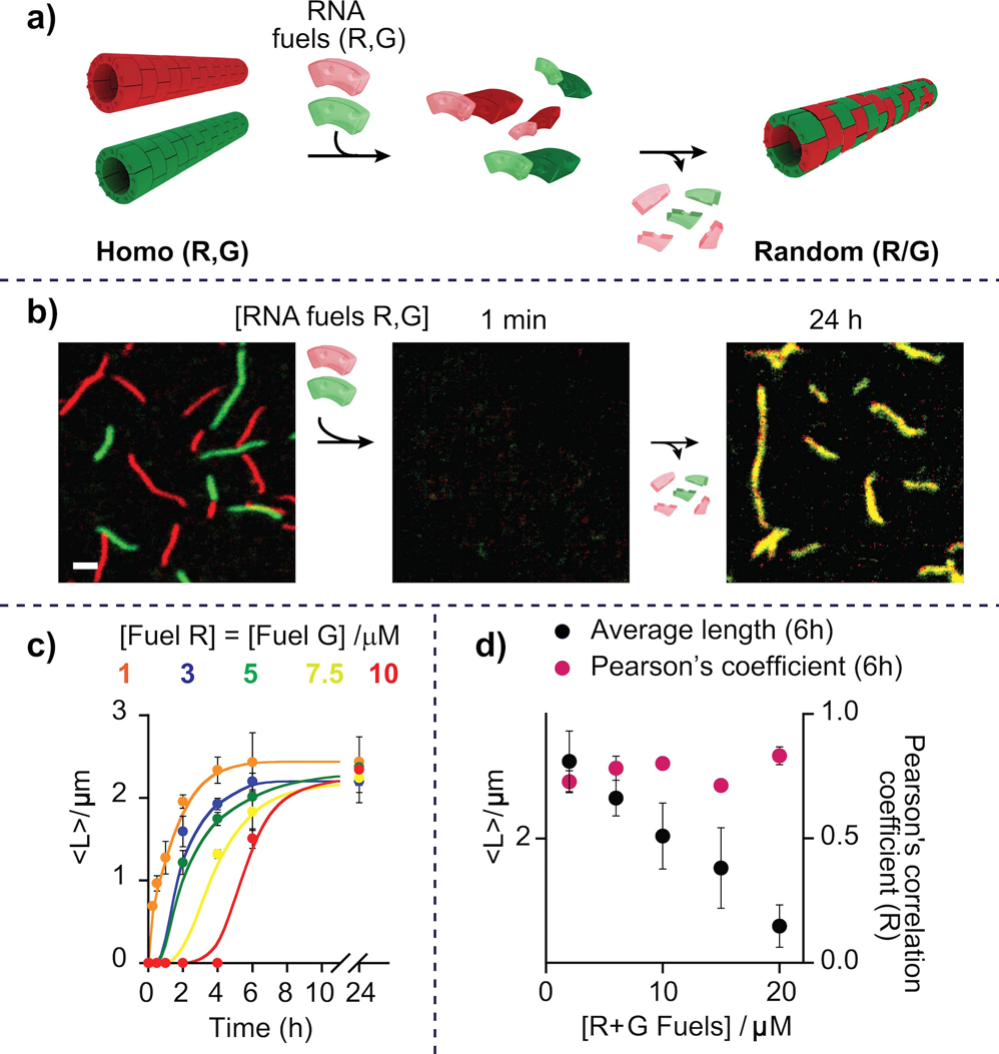
(a) Reorganization from red and green homopolymers to a random (R/G) copolymer driven by RNA fuels. (b) Fluorescence confocal images showing the disassembly of the red (R) and green (G) homopolymers upon the addition of both RNA fuels (R, G) and the autonomous reassembly into R/G random copolymers in the presence of RNase H. Confocal images scale bar, 2.5 μm. (c) Kinetic traces showing autonomous reassembly of R/G random copolymers (average length, ⟨*L*⟩) upon the addition of different concentrations of (R, G) RNA fuels. (d) Average length and Pearson’s coefficient values of reassembled R/G random copolymers after 6 h from fuels addition. Statistical analysis of length distribution is also shown in Figure S5. [tile R] = [tile G] = 0.15 μM, [RNase H] = 30 U/mL, 1 × TAE buffer + 12.5 mM MgCl_2_ + 10 mM DTT, pH 8.0, 25 °C. Error bars represent standard deviation based on triplicate measurements.

In a first series of experiments we demonstrate that homopolymers can be converted to random copolymers with tunable speed (Figure 2 and Figure S2). When annealed separately, red and green tiles assemble into homopolymers that remain stable when mixed together even in the presence of RNase H (Figure S3). The addition of the red and green RNA fuels induces the rapid disassembly of both polymers (no structures observed after 1 min, Figure 2a,b). The enzymatic degradation of the two fuels by RNase H (30 U/mL), however, gradually reactivates the DNA tiles for assembly, but now yielding a tubular structure with both tiles randomly distributed in the assembly (i.e., random R/G copolymer, Figure 2a,b). To quantitatively characterize the spatial localization of distinct tiles in the polymers, we analyzed fluorescence microscopy images of our samples and computed their Pearson’s coefficient (PC), a parameter that estimates the strength of the linear relationship between the fluorescence intensity values of red and green areas: PC values around 0 would indicate low colocalization of fluorophores, while PC values around 1 would indicate high colocalization.^36,37^ For the solution containing homopolymers, we computed a PC of 0.03 ± 0.01, which is in support of a very limited colocalization of the two tiles. In contrast, random copolymers obtained after addition and degradation of the two fuel strands yield a PC value (0.70 ± 0.04) indicating that mixing of the two tiles has occurred.

The speed of reconfiguration from homo to random copolymers can be modulated by varying the concentration of RNA fuel in solution (Figure 2c and Figure S2). To show this, we performed fluorescence confocal microscopy experiments that track over time the transition of red and green homopolymers into random R/G copolymers. Experiments were performed at different concentrations of red and green RNA fuels (Figure 2c, Figures S2 and S4) but at the same fixed concentration (30 U/mL) of fuel-consuming unit (i.e., RNase H). For lower fuel concentrations (i.e., 0.3 and 1 μM) the time required to complete the reconfiguration is in the order of few minutes (Figure 2c,d, and Figure S4). Of note, despite the different reconfiguration kinetics, similar average length values are obtained at 24 h after fuel addition attributed to the fact that the enzymatic reaction has reached completion (Figure 2c). Moreover, because the enzymatic degradation kinetics is similar for the two different fuels, a similar level of colocalization (i.e., average Pearson’s coefficient values = 0.80 ± 0.05) is observed at all times and over the entire fuel concentration range studied (Figure 2d).

**Figure 3:**
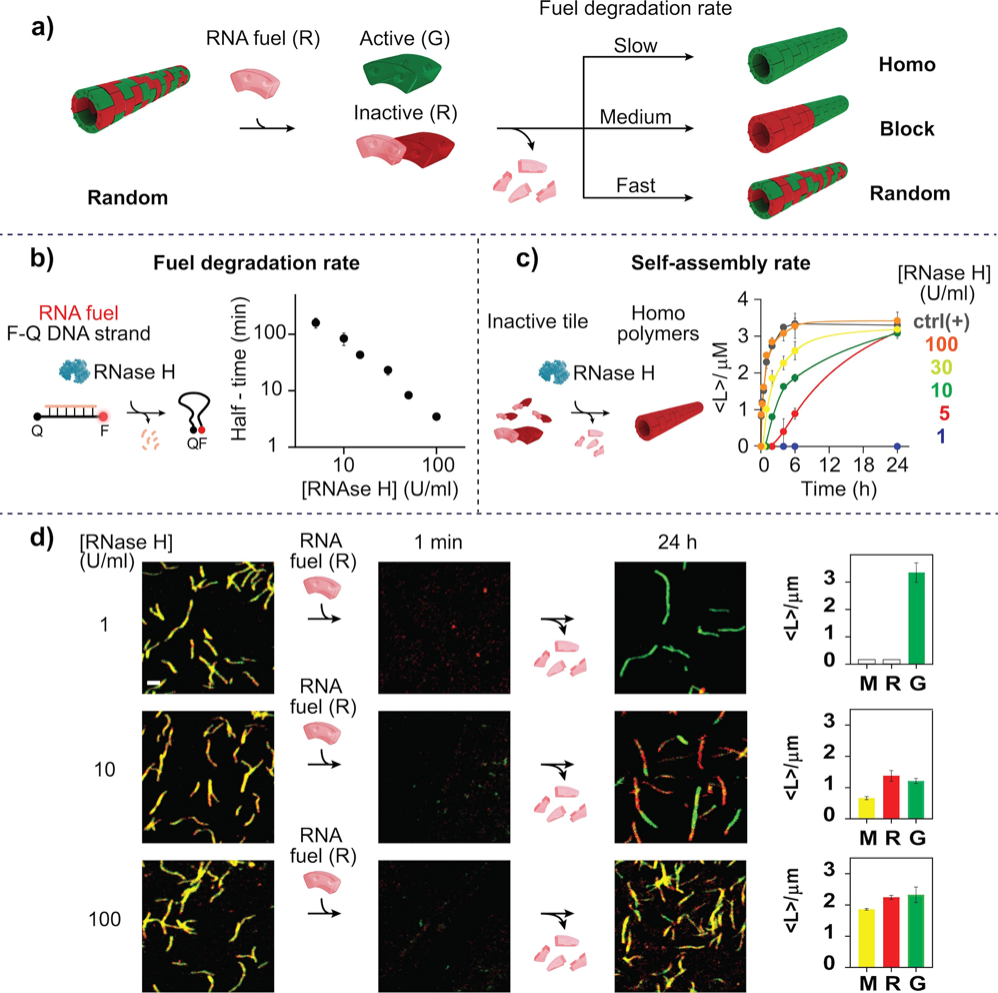
(a) DNA polymers reorganization at different fuel degradation rates. (b) Half-time of RNA fuel at different fuel-consuming unit (RNase H) concentrations. The experiment was performed in the presence of 0.25 μM of F-Q DNA strand and 3 μM of RNA fuel (R). (c) Kinetic traces showing the transient reassembly of inactive DNA tiles by varying the concentration of RNase H. The experiment was performed in the presence of 0.25 μM of red (R) homopolymers inactivated by 3 μM of RNA fuel (R) or 3 μM of control DNA Inhibitor (R). Statistical analysis of length distribution (after 6 h) is also shown in Figure S14. (d) Fluorescence confocal images upon the addition of red RNA fuel at different concentrations of RNase H (1, 10, and 100 U/mL). The average length (⟨*L*⟩/μM) of the structures obtained for each channel (red, R, green, G, and merged, M) after the 24 h reconfiguration is also shown in the bar plot. For the block copolymers each bar column (red and green) represents the average length of each block segment. [tile R] = [tile G] = 0.25 μM, [R fuel] = 3 μM, 1 × TAE buffer + 12.5 mM MgCl_2_ + 10 mM DTT, pH 8.0, 25 °C. Confocal images scale bar: 2.5 μm. Error bars represent standard deviation based on triplicate measurements.

A key element of our approach is that by supplying specific fuel molecules and by regulating their degradation rate—which is done by simply varying the amount of RNase H—it is possible to temporarily exclude specific tiles from participating in the self-assembly process. This feature can be used to route multiple polymers reconfiguration pathways and to control their kinetics. For example, starting from random copolymers, the addition of a single RNA fuel will cause a single tile to become inactive, while the other type of tile can reassemble. Depending on how fast the inactive tile is reactivated by RNA degradation, the system is qualitatively expected to yield homopolymers, block, or random copolymers respectively for a degradation rate that is slow, medium, or fast in relation to the rate of tile assembly (Figure 3a). Importantly, of these transitions in particular the transition from random to block copolymer is of interest as it implies the fuel-triggered population of an entropically disfavored state.

As these reconfiguration pathways critically depend on enzyme speed, we first determined the kinetics of fuel degradation by RNase H. We studied this via time-course experiments with an RNA fuel (3 μM) hybridized to a fluorescent-labeled DNA strand (0.25 μM), following the fluorescent signal after the addition of different concentrations of RNase H (Figure 3b and Figure S6). The half-time of RNA fuel degradation increases from 3.5 to 160 min as the concentration of RNase H decreases from 100 to 5 U/mL (Figure 3b). These results are reproduced by using a simple ordinary differential equation (ODE) model that captures hybridization of the RNA fuel and a dual-labeled DNA probe and RNase H degradation. By fitting the model (Figures S7 and S8) to the data, we found hybridization and enzyme kinetic parameters comparable with previous estimates in the literature.^38,39^

Next, we asked whether we could control the rate of tile assembly (into homopolymers) in the range of RNase H concentrations considered for the duplex control experiments (100–5 U/mL). Using a computational model for tile assembly in the presence of RNA fuel and RNase H, we found that the half-time for assembling 0.25 μM tiles can be tuned between a few minutes at 100 U/mL RNase H and more than 10 h at 1 U/mL (Figures S9 and S10). It should be noted that this unfitted model underestimates the kinetics of nanotube growth because it captures the process of tile assembly but does not model the average nanotube length and how it is affected by slower joining and ripening.^31^ In addition, the model does not take into account incomplete RNA degradation products that may slow down tile assembly.^25^ Yet, these predictions suggest that RNase H level in the range 1–100 U/mL can yield dramatically different kinetics of tile assembly. This expectation is met by experiments in which we measured the mean nanotube length when tiles are reactivated by RNase H: at high levels of RNase H (100 U/mL) tiles reactivate fast, with nanotubes reaching their half-max length in *t*_1/2_ = 32 min, a time scale that is comparable to that achieved by adding a DNA activator that removes the fuel from tiles through a strand displacement reaction (Figure 3c, Figures S11 and S12).^34,26^ Under these conditions, the tubular structure mean length levels off after only 4 h. At medium RNase H levels (10 U/mL), tiles reactivation is slower (*t*_1/2_ = 253 min), and the nanotube mean length levels off after 24 h. Finally, at low levels of RNase H (1 U/mL), tubular structures start forming after more than 48 h, and we did not observe significant growth over the course of our experiment (likely due to loss of enzyme activity) (Figure S13).

Having identified levels of RNase H that yield slow, medium, and fast tile reassembly, we sought to demonstrate kinetically controlled reconfiguration using the corresponding three RNase H concentrations (1, 10, and 100 U/mL). To do so, we started from random R/G copolymers, obtained by mixing in the same solution green and red active tiles. The addition of only the red RNA fuel results in the rapid disassembly of the whole structure under all the experimental conditions tested. This is due to the fact that the red and green tiles are randomly distributed in the tubular structure, and the fuel-triggered deactivation of one tile induces the collapse of the entire structure.^35^ At a slow fuel degradation rate the reactivation of red tiles is not achieved over the 24 h course of the experiment, and thus the disassembled green tiles that remain in solution in an active conformation spontaneously reassemble into the green homopolymer (Figure 3d). At medium fuel degradation rate, formation of the green homopolymer is still faster than the activation of the red tiles, so red polymers self-assemble at the two ends of the green homopolymers, leading to an ordered block copolymer (Figure 3d). It should be noted that the process of block copolymers formation is not deterministic and could lead to different types of block polymers (RG or RGR). Finally, at fast fuel degradation rate, the red tiles are rapidly activated soon after fuel addition and coassemble together with the green tiles to re-form random polymers. Statistical analysis of the microscopy images confirms the modulation of the transient reconfiguration achieved at different concentrations of enzyme (Figure 3d, bar plot). For example, the average length (⟨*L*⟩) of the tubes formed after reconfiguration at a medium fuel degradation rate appears similar for the green and red tubes. However, the yield of randomly distributed random structures (observed with the merged channel, M) is quite low (PC = 0.40 ± 0.03). On the contrary, at fast fuel degradation rate the average length of both green and red structures is very similar to that of the random R/G structures (see M channel length), and Person’s coefficient (PC = 0.80 ± 0.06) confirms the random distribution of tiles.

**Figure 4:**
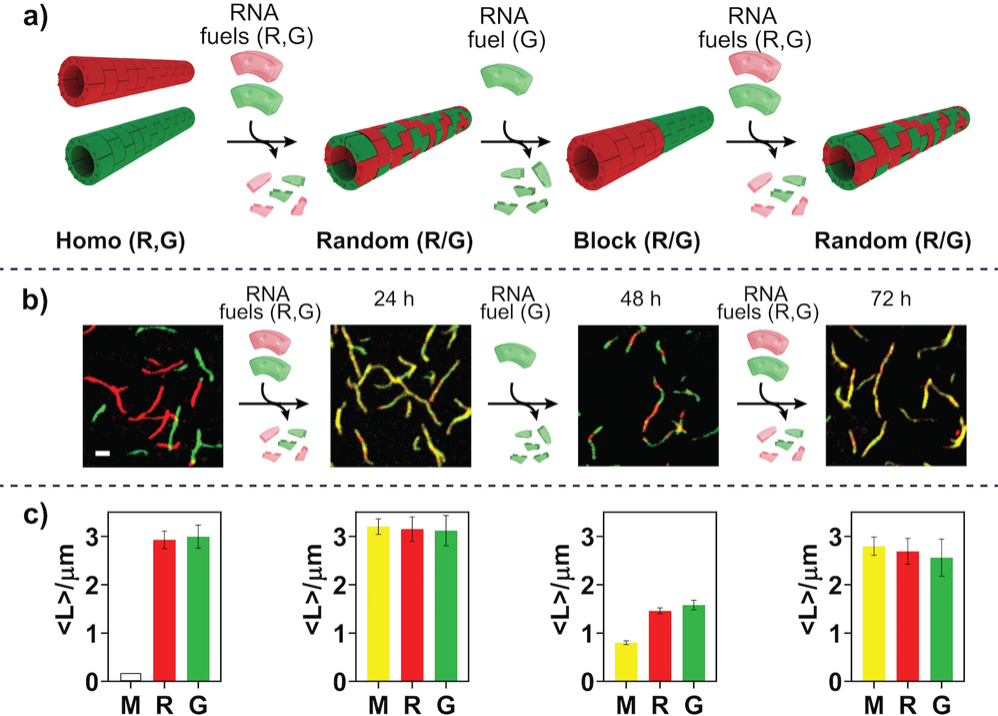
(a) Multiple reorganization cycles of DNA polymers upon the repetitive additions of different RNA fuels. (b) Fluorescence confocal images after each reorganization cycle. (c) Average length values of DNA-based polymers before and after each cycle of reconfiguration obtained for each channel (red, R, green, G, and merged, M). For the block copolymers each bar column (red and green) represents the average length of each block segment. The experiments here were performed in the presence of 0.15 μM of preformed red (R) and green (G) homopolymers. First and third reconfigurations were obtained by adding both red and green fuels (0.3 μM). The second reconfiguration was obtained by adding only the green fuel (1.0 μM). Experiments performed in 1 × TAE buffer + 12.5 mM MgCl_2_ + 10 mM DTT + 30 U/mL RNase H, pH 8.0, 25 °C. Confocal images scale bar: 2.5 μm. Error bars represent standard deviation based on triplicate measurements.

The fuel-triggered polymers reorganization is fully reversible and can be repeated multiple times by sequentially supplying fuel molecules over time. To demonstrate this, we have started with a solution containing two separate red and green homopolymers (PC = 0.02 ± 0.01) and an RNase H concentration to install medium fuel-degradation. Upon the addition of both fuels we observed the rapid disassembly and, after fuels degradation, the reassembly of the structures into random copolymers (as both fuels are degraded at the same rate) (PC = 0.80 ± 0.06) (Figure 4a,b). The addition of a new aliquot of only the green fuel to the same solution induces the disassembly of the entire structure (as discussed previously). However, since the red tiles remain active, they self-assemble into red homopolymers, and only after green fuel degradation we observe the formation of block copolymers (PC = 0.50 ± 0.02) (Figure 4a,b). In the last reaction cycle, we have then added a new aliquot of both fuels (red and green) and achieved another reorganization back into the random structures (PC = 0.80 ± 0.01) (Figure 4a,b). Also in this case, the analysis of the confocal images in terms of average length of green (G channel), red (R channel), and random (merged, M, channel) structures (Figure 4c) and PC values supports the described reconfiguration.

A similar efficient reversibility in successive reconfigurations was also observed by repetitive additions of the red and green fuels (Figure S15), leading to transient disassembly and formation of random R/G structures with average length and PC values indistinguishable from one another over three consecutive reaction cycles (PC_24h_ = 0.80 ± 0.03, PC_48h_ = 0.90 ± 0.01, and PC_72h_ = 0.80 ± 0.03).

Finally, to demonstrate the versatility of our approach and to show that the strategy can also function in systems of higher complexity, we have also performed preliminary studies employing three orthogonal tiles (red, green, and blue) addressable with three different fuel RNA strands and each labeled with a different fluorophore. Also in this case, spontaneous reorganization can be achieved by starting from a mixture of the three homopolymers and adding all three fuel RNA strands leading to the transient disassembly of all structures and the concomitant activation of the three tiles that self-assemble into a random polymer with a statistical distribution of the three tiles. The reaction cycle also in this case is reversible, leading to similar lengths of the assembled random polymers over two consecutive additions of fuels (Figure S16).

## Conclusion

We have shown a new strategy that allows the chemically fueled spontaneous reconfiguration of self-assembled DNA polymers. The strategy relies on the transient deactivation of building blocks by RNA fuels that subtract them temporarily from participation in the self-assembly process. By simply controlling the rate at which the building blocks are reactivated, it is possible to determine the composition of the polymer. Importantly, we have shown a mechanism to use RNA fuels and enzymes that degrade them to induce the spontaneous conversion of a disordered polymer into a higher order polymer, which is disfavored from a thermodynamic point of view. Similar reorganization mechanisms could be applied to other DNA-based structures that are assembled/disassembled under isothermal conditions by using different inputs.^40−44^

Considering the possibility to introduce multiple, sequence distinct fuel-recognition sites, and the possibility to decorate the polymer building-blocks with different functional ligands, we anticipate that this strategy will be useful for the development of multifunctional systems in which the output is determined by the relative organization of the functional groups in the polymer. The reorganization of different biomolecules attached on a cargo-delivery DNA structure^45−47^ could, for example, lead to a different targeting ability of the structure itself. Similarly, the possibility to control the distribution of metal particles could yield DNA structure with reconfigurable optical properties.^48,49^

## Supporting Information Available

Additional experimental details, method, and DNA sequences used; description of the kinetic model used in the work; additional experiments including reconfiguration, statistical analysis, and enzyme kinetic study (PDF)

## Dedication

This paper is dedicated to Prof. Ned Seeman, whose visionary ideas marked the beginning of DNA nanotechnology.
