# Supplementary material for: Spontaneous Reorganization of DNA-Based Polymers in Higher Ordered Structures Fueled by RNA
Source: J Am Chem Soc. 2021 Nov 29;143(48):20296–301. doi: 10.1021/jacs.1c09503 (PMC8662731; doi:10.1021/jacs.1c09503)
Supplement: Supplementary file 1 — ja1c09503_si_001.pdf [file ja1c09503_si_001.pdf]

Spontaneous reorganization of DNA-based polymers in higher ordered structures fueled by RNA

Serena Gentile, Erica Del Grosso, Passa E. Pungchai, Elisa Franco, Leonard Prins,* Francesco Ricci*

Supporting Information:

EXPERIMENTAL PROCEDURES

Chemicals

Reagent-grade chemicals (DEPC-treated water, Na_2HPO_4, Trizma hydrochloride, Trizma base, Acetic acid, EDTA, KCl, $MgCl_2$, 1,4-Dithiothreitol) were purchased from Sigma-Aldrich (St. Louis, Missouri, USA) and used without further purifications. RNase H recombinant was purchased from New England BioLabs.

DNA oligonucleotides

HPLC-purified DNA and RNA oligonucleotides were purchased from Biosearch Technologies (Risskov, Denmark), Metabion International AG (Planegg, Germany) and employed without further purifications. The DNA oligonucleotides were dissolved in phosphate buffer 50 mM, pH 7.0 and stored at -20 °C until use. The RNA oligonucleotides were dissolved in DEPC-treated water and stored at -20 °C until use.

DNA-based polymer self-assembly

The DNA-based structures were prepared as reported elsewhere.[1,2] Briefly, we employed a version of DAE-E tile originally reported by Winfree, Rothemund and Franco.[1-4]

Following the strategy reported in our previous work,[5] we have re-designed three different DNA tiles containing the same 5-nt sticky ends portion responsible for self-assembly to include a different fuel binding domain that can be specifically addressed by RNA fuel strands which allow to deactivate their capacity to self-assemble.[3-5] The formation of individual tiles requires thermal annealing, however, DNA tubular structures self-assembly proceeds at room temperature. Before use, RNase H was previously activated by incubation for 40 min at 37°C in Tris-HCl/K^+/Mg^{2+} buffer (50 mM Tris-HCl, 50 mM KCl, 3 mM $MgCl_2$) in presence of 50 mM DTT at pH 8. All experiments unless otherwise noted were obtained in a Tris Acetate-EDTA (TAE)/Mg^{2+} (TAE) buffer 1x, 12.5 mM $MgCl_2$ at pH 8 at 25°C.

The detailed protocols for each reorganization are reported in spontaneous reorganization of DNA-based polymers section.

Fluorescence imaging of DNA polymeric structures

For fluorescence microscopy imaging the central strand of each tile (S3, see sequence below) was labeled at 5' end with a different fluorophore (Quasar570, Quasar670 or Atto488). A confocal laser scanning microscope Olympus FV-1000 was used to monitor the reconfiguration experiments. The emitted photons were collected by a 60x, oil objective. The solutions containing the DNA-based polymers were diluted to achieve a final concentration of 50 nM. A 10 µL drop of this diluted solution was then deposited between a clean microscope slide and a coverslip and the images at three different wavelengths (Laser 488 Argon λ_{ex} = 488 nm, λ_{em} = 520 nm ; Laser 543 HeNe λ_{ex} = 543 nm, λ_{em} = 572 nm; Laser 635 Diodo λ_{ex} = 635 nm, λ_{em} = 668 nm) were taken.

To investigate the speed of DNA tiles assembly into homopolymers (Figures 3c, S3, S4, S12 and S13b) an Axio Scope A1 ZEISS microscope was used. The emitted photons were

collected by a 100x oil objective and a monochrome CCD camera (Axiocam 503 mono - ZEISS).

The images were analyzed using ZEN 2 lite (ZEISS) software. All the samples were imaged at 50 nM tile concentration in the corresponding experimental condition (Figures 3c, S3, S4, S12 and S13b). A single drop (2.5 μL) of the samples was placed between a microscope glass slides and a coverslip and imaged using a Cy3 filter set (EX: 530-550 nm; BP: 565 nm; EM: 575-635).

DNA-based structure average length distributions were quantified by image metrology using the SPIP software (www.imagemet.com). R/G images (red and green merged channels) were obtained by firstly analyzing the structures where colocalization of the red and green tiles occurs by using a plugin for ImageJ software named Colocalization and then analyzed using the SPIP software described above. Colocalization analysis were performed by using a plugin for ImageJ software named JACoP -Just Another Co-localization Plugin (threshold = 50).[6,7].

Fluorescence experiments

Time-course fluorescence measurements (Figures 3b, S6 and S13a) were carried out on a Cary Eclipse Fluorimeter (Varian) using a quartz cuvette of reduced volume (100 μL).Working wavelengths were set to λ_{ex} = 640 (± 5) nm and λ_{em} = 670 (± 10) nm monitor the Cy5 fluorescence signal. The duplex was formed by hybridizing 0.25 μM of dual-labeled linear DNA strand probe (DNA F-Q strand) with 3 μM of RNA fuel (R) in 1 × TAE pH 8.0 supplemented with [Mg^{2+}] = 12.5 mM and incubating at T = 25° C for 30 min. Duplex fluorescence intensity was recorded for 20 minutes until complete stabilization of the Cy5 fluorescence signal before addition of different concentrations of RNase H (1, 5, 10, 15, 30, 50, 100 U/ml) (Figure S6).

It should be noted that for low levels of RNase H (1 U/ml) a significant variation of the signal is observed for times greater than 24 h (Figure S13). The experiment was followed with the same protocol reported above but after 48h an aliquot of 5 mM DTT was added into the solution to preserve the RNase H activity during the entire course of experiment.

DNA strands for polymer assembly

The following sequences are the DNA strands used to form the addressable tiles (S1-S5). Nucleotides in italics for strands S2 and S4 denote the sticky end portions. Strand S3 has been used conjugated to a different fluorophore for each tile at the 5’ end. Strand S2 also contains the 7-nt fuel binding domain (in bold) that is different for each addressable tile. [3-5] The RNA fuel binds to S2 through a 14-nt portion that first binds to the 7-nt fuel binding domain (in bold) of S2 and then invades the 5-nt sticky end (in italics) and 2 additional nucleotides (underlined). Sequences and modification schemes for DNA tiles and RNA fuel are reported below:

Red Tile:

**Name**	**Sequence**
S1	5’-CTC AGT GGA CAG CCG TTC TGG AGC GTT GGA CGA AAC T-3’
S2_R	5’-**TGG TAT T** *GTC TG* GTA GAG CAC CAC TGA G *AGG TA*-3’
S3_Q570	5’-Q570-TCC AGA ACG GCT GTG GCT AAA CAG TAA CCG AAG CAC CAA CGC T-3’
S4	5’-*CAG AC* AGT TTC GTG GTC ATC G *TACCT*-3’
S5	5’-CGA TGA CCT GCT TCG GTT ACT GTT TAG CCT GCT CTA C-3’
RNA fuel (R)	5’-AC*C AGA C***AA UAC CA**-3’
Control DNA fuel (R)	5’-ACC AGA CAA TAC CAA TCC GC-3’
Control DNA Activator (R)	5’-GCGGATTGGTATTGTCTGGT-3’

Green Tile:

Name	Sequence
S1	5’-CTC AGT GGA CAG CCG TTC TGG AGC GTT GGA CGA AAC T-3’
S2_G	5’-**CTT ACG T** *GTC TG* GTA GAG CAC CAC TGA G *AGG TA*-3’
S3_Q670	5’-Q670-TCC AGA ACG GCT GTG GCT AAA CAG TAA CCG AAG CAC CAA CGC T-3’
S4	5’-*CAG AC* AGT TTC GTG GTC ATC G *TACCT*-3’
S5	5’-CGA TGA CCT GCT TCG GTT ACT GTT TAG CCT GCT CTA C-3’
RNA fuel (G)	5’-AC*C AGA C***AC GUAAG**-3’

Blue Tile:

Name	Sequence
S1	5’-CTC AGT GGA CAG CCG TTC TGG AGC GTT GGA CGA AAC T-3’
S2_B	5’-**AGT TCA A** *GTC TG* GTA GAG CAC CAC TGA G *AGG TA*-3’
S3_Atto488	5’-Atto488-TCC AGA ACG GCT GTG GCT AAA CAG TAA CCG AAG CAC CAA CGC T-3’
S4	5’-*CAG AC* AGT TTC GTG GTC ATC G *TACCT*-3’
S5	5’-CGA TGA CCT GCT TCG GTT ACT GTT TAG CCT GCT CTA C-3’
RNA Fuel (B)	5’-AC*CAGAC***UUGAACU**-3’

Protector strands:

Name	Sequence
P1	5’-TAC CTC TCA GTG GAC AGC CG-3’
P2	5’-GCG TTG GAC GAA ACT GTC TG-3’

Strands for fluorescence experiments (Figure 3, Panel b):

Name	Sequence
DNA F-Q strand	5'-BHQ-2 GCG GAT TGG TAT TGT CTG GT- Cy5-3'
RNA fuel (R)	5’-AC*C AGA C***AA UAC CA** -3’

Spontaneous reorganization of DNA-based polymers

For the RNA-triggered reorganization from red (R) and green (G) homopolymers to a random R/G co-polymer with tunable speed (Figures 2 and S2) we have first prepared the two homopolymers separately. To do so the five strands of each tile were mixed at a concentration of 5 µM (tile concentration= 5 µM) in H_2O/Mg^{2+} (12.5 mM $MgCl_2$) and annealed with a Bio-Rad Mastercycler Gradient thermocycler by heating to 90°C, and cooling to 20°C at a constant rate over a 6-hour period. The so formed homopolymers were then mixed to a final concentration of each tile of 0.15 µM using a buffer solution containing 30 U/ml RNase H and 10 mM DTT. Both red and green RNA fuels (red, R and green, G) were then added at different concentrations in each solution and the modulation of the kinetic reassembly into random structures was followed during time (1 min, 2, 4, 6, 24 hours) with a confocal microscopy at final concentration of 50 nM.

For the kinetic modulation of the DNA tiles assembly into homopolymers (Figures 3c, S12 and S13b) the red homopolymers were prepared by the annealing procedure reported above. The so formed homopolymer solution was diluted to a final concentration of 0.25 µM in a buffer solutions (1 × TAE buffer + 12.5 mM $MgCl_2$ at pH 8.0, 25 °C, 10 mM DTT) and 3 µM of RNA fuel was added. Then, the addition of different concentrations of RNase H (0, 1, 5, 10, 30, 100 U/ml) to the inactive tiles solution causes the reactivation of the tiles and the reassembly of the DNA homopolymers. A positive control was performed by adding the DNA control activator (6 µM) to the inactive tiles solution containing 0.25 µM of tiles and 3 µM of DNA (Figures S11 and S12) [3,4]. All the assembly experiments were followed during time by using an Axio Scope A1 ZEISS microscope at final concentration of 50 nM (Figures S12 and S13b). For low levels of RNase H (1 U/ml) the assembly requires a period longer than 24h (Figure S13). After 48h 5 mM DTT was added into the

solution to preserve the RNase H activity during the entire course of experiment (Figure S13).

For the DNA-based polymer reorganization from R/G random co-polymer to G homopolymer, R/G block co-polymer or R/G random co-polymer (Figure 3d) at different fuel degradation rates, we have prepared two separate solutions of the green and red protected DAE-E tiles. Each protected tile was first formed by mixing six different DNA strands (four strands of the tile + two protector strands)[2] at 5 μM in H_2O/Mg^{2+} (12.5 mM $MgCl_2$) and annealing with a Bio-Rad Mastercycler Gradient thermocycler as reported above. The deprotector strand (2 μM) was added to a solution containing 1 μM of both green and red protected tiles allowing the activation to occur for 16 hours at 25°C. The solution of the so-formed R/G random co-polymers was then diluted to a final concentration of each tile of 0.25 μM in a buffer solutions containing different concentrations of RNase H (1, 10, 100 U/ml), 10 mM DTT and 3 μM of RNA fuel was added to each solution 30 min after the addition of RNase H and DTT. Confocal images were taken after 1 min and 24 hours using a diluted solution (final tile concentration = 50 nM).

To test the reversibility of our system, multiple reorganization cycles of DNA polymers by using two- and three-tiles were carried out with repetitive additions of different RNA fuels (Figures 4, S15 and S16).

For multiple reorganization cycles from R and G homopolymers to R/G random co-polymer (Figure 4) by going through R/G block co-polymers we have prepared two separate solutions of the green and red DNA-based homopolymers as reported above. The red and green homopolymer solution was then diluted at final concentration of each of 0.15 μM

using a buffer solution containing 30 U/ml RNase H and 10 mM DTT. Confocal images were taken at 50 nM concentration before and after each cyclic addition of RNA red R and green G fuels. More specifically, 0.3 μM of both RNA fuels were firstly added to the homopolymers solution. The addition of both fuels causes the rapid disassembly of both DNA-based homopolymers (image taken 1 min after) and the reassembly into R/G random co-polymers (image taken after 24h). A second aliquot of 1 μM of green (G) RNA fuel was then added to the same solution of re-assembled R/G random co-polymers. The addition of only one fuel allows to disassemble the entire structure of the polymers within 1 min. Confocal images taken after 48h showed the reassembly into R/G block co-polymers. The third cycle started after the addition of 0.3 μM of red and green RNA fuels into the same solution. The disassembly (1 min) and the re-assembly again into R/G random co-polymers after 72h was then followed with the confocal microscope as reported above.

For the multiple reorganization cycles from R and G homopolymers to R/G random co-polymer (Figure S15) we have prepared two separate solutions of the green and red DNA-based homopolymers as reported above. The two homopolymer solutions were then diluted and mixed at final concentration of 0.15 μM using a buffer solution containing 30 U/ml RNase H and 10 mM DTT. Three reconfiguration cycles were performed by adding red and green RNA fuels every 24h. Confocal images were taken at 50 nM concentration before and after each cyclic addition of both RNA fuels. More specifically, 0.3 μM of both fuels were firstly added to the homopolymers solution. A second and third aliquots of red and green fuels (0.3 μM) were then added following the same reversible behavior observed in the first cycle. For the second and third cycles confocal images were taken 1 min and 48 and 72 hours, respectively (Figure S15).

For the three-component cyclic reorganization from R, G, B homopolymers to R/G/B random co-polymer we have prepared the three homopolymers separately and performed the same protocol described above (Figure S16).

Kinetic models

Degradation of RNA fuel bound to a dual-labeled linear DNA strand probe

We model the experiments described in Fig. 3b using the following equivalent chemical reactions. The interaction between dual-labeled linear DNA strand probe and RNA fuel is modeled with a second order reaction that yields a duplex:

$$DNA + RNA\,fuel \xrightarrow{k_h} Duplex$$

The duplex becomes a binding site for RNase H, which degrades the fuel bound to DNA and releases the dual-labeled linear DNA strand probe.

$$RNase\ H + Duplex \underset{k-,H}{\overset{k+,H}{\Longleftrightarrow}} RNase\ H \cdot Duplex \xrightarrow{K_{CAT,H}} DNA$$

We use the Michaelis-Menten quasi steady-state approximation to simplify the enzyme-substrate reactions, and we write the following equation to describe the level of free RNase H:

$$[RNase\ H] = \frac{[RNase\ H]_0}{1 + \frac{[Duplex]}{K_M}}$$

Where $[RNase\ H]_0$ is the total concentration of RNase H (which remains constant). The kinetics of dual-labeled linear DNA strand probe – RNA fuel binding and RNase H degradation are then modeled using the following ordinary differential equations (ODEs):

$$\frac{d\,[DNA]}{dt} = -k_h[DNA][RNA\,fuel] + \frac{k_{cat}}{K_M}[RNase\,H][Duplex] \quad (1)$$

$$\frac{d\,[RNA\,fuel]}{dt} = -k_h[DNA][RNA\,fuel] \quad (2)$$

$$\frac{d\,[Duplex]}{dt} = k_h[DNA][RNA\,fuel] - \frac{k_{cat}}{K_M}[RNase\,H][Duplex] \quad (3)$$

These ODEs can be computationally integrated using MATLAB's ode23s solver. This model does not account for potential loss of RNase H activity over time and neglects the build up of incomplete degradation products that may slow down the overall recovery of duplex. [8,9]

Assembly of DNA tiles in the presence of RNA fuel inactivating the tiles and RNase H

We built a simple model to recapitulate the kinetics of tile assembly shown in Fig. 3c. Our model is based on the computational models.[10,2] We assume that active DNA tiles become inactive when bound to RNA fuel:

$$Active\ tile + RNA\ fuel \xrightarrow{k_h} Inactive\ tile$$

Inactive tiles are activated when RNase H degrades RNA Fuel that is part of the Inactive tile complex.

$$RNase\ H + Inactive\ Tile \underset{k-,H}{\overset{k+,H}{\Longleftrightarrow}} RNase\ H \cdot Inactive\ Tile \xrightarrow{K_{CAT,H}} Active\ Tile + RNase\ H + Waste$$

We again use Michaelis-Menten assumptions to describe the enzyme-substrate reaction, so we describe the level of free RNase H using this expression:

$$[RNase\ H] = \frac{[RNase\ H]_0}{1 + \frac{[Inactive\ Tile]}{K_M}}$$

Like before, $[RNase\ H]_0$ is the total (constant) concentration of RNase H. We model nucleation and elongation of tiles according to Zhang, *et al*. [2]

$$n \cdot \; Active\ Tile \xrightarrow{k_{nuc}} Nucleus$$

$$Nucleus + Active\ Tile \xrightarrow{k_{elong}} Nanotube$$

We then derive the following ODEs from the chemical reactions:

$$\frac{d\ [Inactive\ Tile]}{dt} = +\ k_h[Active\ Tile][RNA\ fuel] - \frac{k_{cat}}{K_M}[RNase\ H][Inactive\ Tile] \quad (4)$$

$$\frac{d\ [Active\ Tile]}{dt} = \ -k_h[Active\ Tile][RNA\ fuel] -$$

$$-nk_{nuc}[Tile]^n - k_{elong}[Tile][Nanotube] + \\ + \frac{k_{cat}}{K_M}[RNase\ H][Inactive\ Tile] \qquad (5)$$

$$\frac{d\ [RNA\ fuel]}{dt} = \ -k_h[Active\ Tile][RNA\ fuel] \quad (6)$$

$$\frac{d\ [Nucleus]}{dt} = \ nk_{nuc}[Active\ Tile]^n \quad (7)$$

Parameter fitting

We fitted the parameters of model (1)—(3) to the kinetic data in SI Fig S6 using MATLAB's `fmincon` routine. Specifically, we fitted the hybridization rate constant k_h, the dissociation parameter K_M and the catalytic rate k_{cat}. The fitting bounds and the best fitted value are reported in the table below. The fitting procedure was repeated starting from 100 initial conditions randomly selected in the interval 0.1-10 times a set of nominal values k_h=5 10^3/M/s, K_M =150 10^{-9} M, and k_{cat}=0.1 /s. Nominal values were chosen based on previous work, see for example Agarwal et al. 2021. [10] Because the RNase H concentration is not provided from the vendor, we also fitted a factor to convert the U/mL used in every experiment to units of molar. The conversion factor was fitted with lower bound 0 and upper bound 10^{-6} , with initial condition randomly selected in the range 0.1-10 times a nominal value of 10^{-9}. The best fitted conversion factor is 0.43 10^{-9}, which indicates that one U/mL of RNase H corresponds to an estimated 0.43 nanomolar concentration of

RNase H. Notably, because the conversion factor has to be multiplied by k_{cat} in ODEs (1)—(3), there is a numerical tradeoff in the obtained best fits.

Parameter	Units	Lower Bound	Upper Bound	Best fitted value
k_h	1/M/s	0	10^6	$6.59\ 10^3$
K_M	M	0	10^{-6}	$189.1\ 10^{-9}$
k_{cat}	/s	0	1	0.24

Table S1. Best fitted parameters for model (1)-(3).

The parameters in the table above, together with the conversion factor of 0.43 nM, were used to estimate the assembly kinetics in model (4)—(8) without further fitting.

Computational simulations showing the solutions of ODE models (1)—(3) and (4)—(8) are in the next figures.

Supplementary Figures

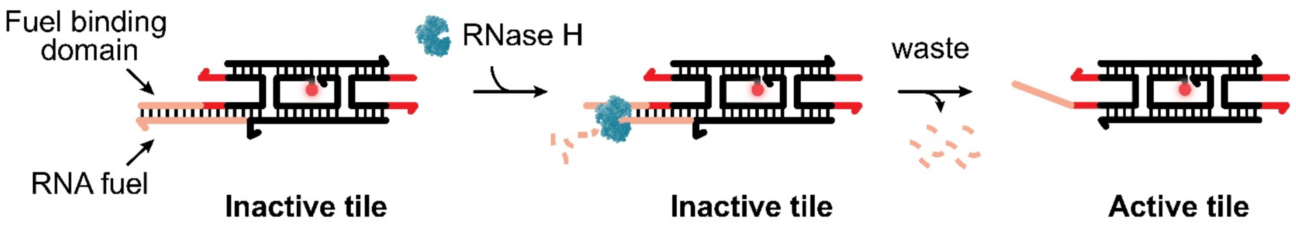

Figure S1. *Scheme showing the tile activation in presence of RNase H. An RNA fuel (light red) acts as an invader strand that binds the 7-nt fuel binding domain of the tile (light red portion) and blocks one of the sticky ends leading to DNA tiles inactivation (and tubes disassembly). The RNase H enzyme selectively degrades the RNA fuel when is bound to the DNA-tile. The degradation of the RNA fuel induces the re-activation of the tile and the re-assembly of the polymeric structure.*

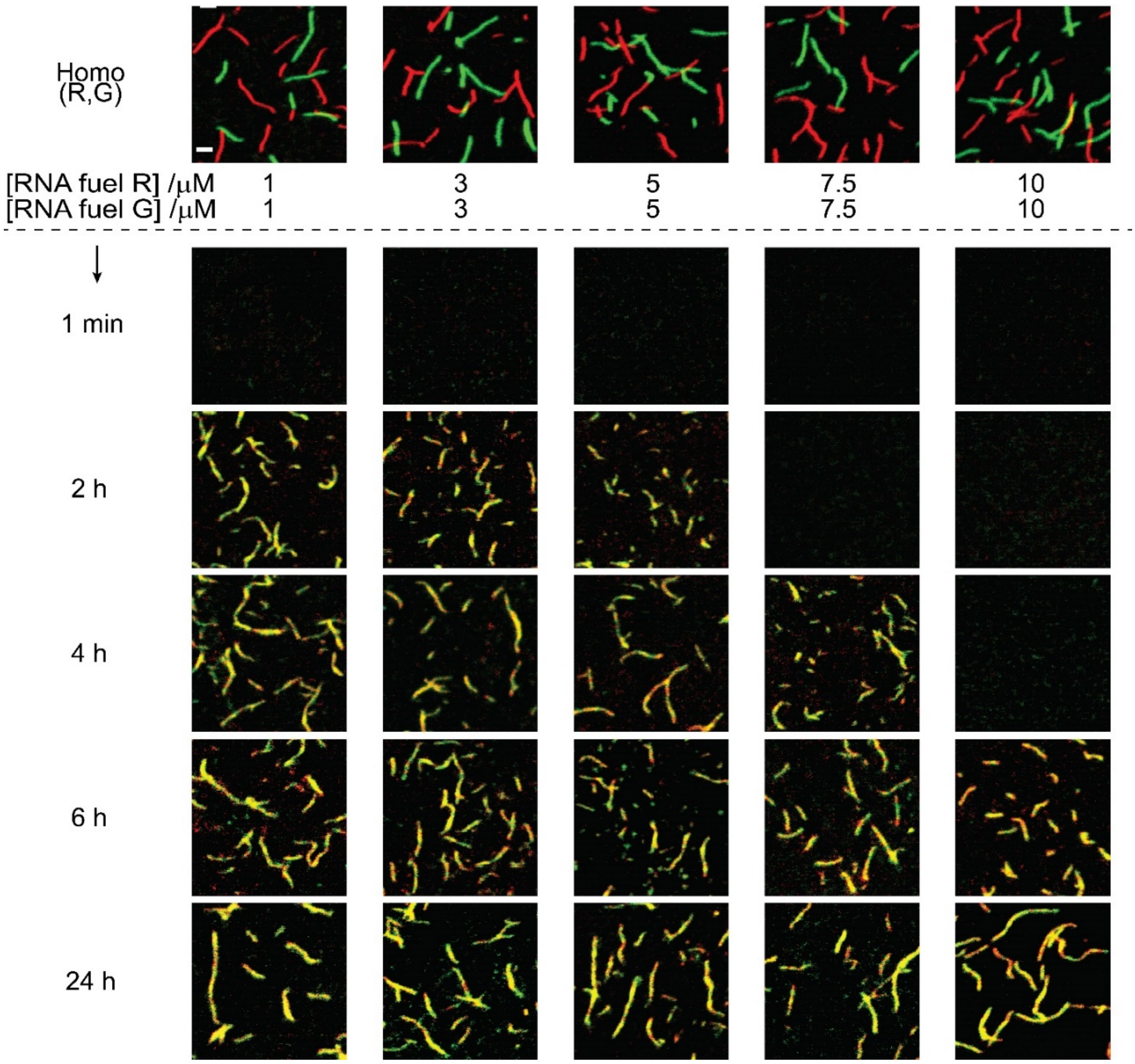

Figure S2. *RNA-triggered reorganization from red (R) and green (G) homopolymers to a random R/G co-polymer with tunable speed. Fluorescence confocal images show the disassembly of the R and G homopolymer upon the addition of different concentrations of both RNA fuels (R,G) (indicated in each column) and the reassembly into R/G random co-polymers in presence of RNase H (30 U/ml). Experimental conditions are the same described in Figure 2. Confocal images scale bar, 2.5 μm.*

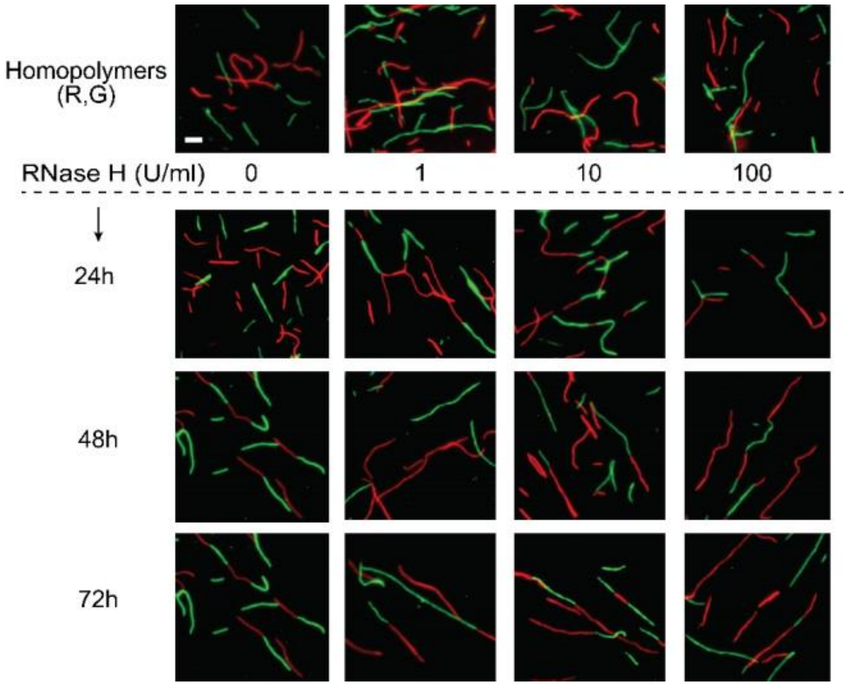

Figure S3. *Fluorescence images showing the stability of DNA nanostructures at different concentrations of RNase H (1, 10, 100 U/ml). The presence of the enzyme does not affect the integrity of the structures that remain stable for the entire time range of experiments (72 h). The experiments shown in this figure were performed in presence of 0.15 µM of red (R) and green (G) homopolymers in 1 × TAE buffer + 12.5 mM* $MgCl_2$ *+ 10 mM DTT at pH 8.0, 25 °C. Concentrations of RNase H tested are indicated in the figure. Fluorescence microscopy images scale bar, 2.5 µm.*

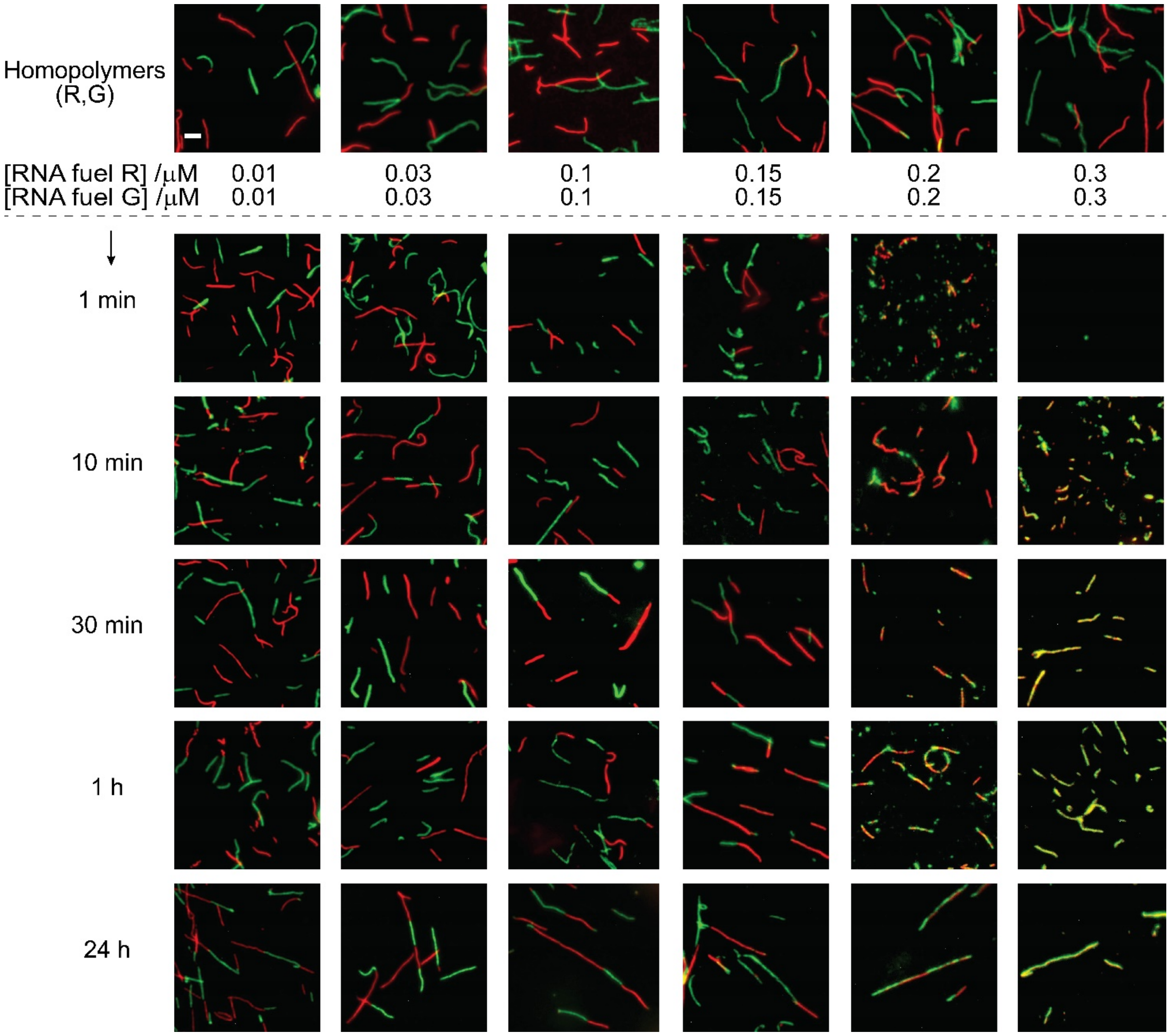

Figure S4. *Fluorescence images showing the disassembly process of DNA-based red (R) and green (G) homopolymers upon the addition of concentrations of both RNA fuels (R,G) lower than those used in Figures 2, S2. The experiments shown in this figure were performed in presence of 0.15 µM of red (R) and green (G) homopolymers and RNase H (30 U/ml) in 1 × TAE buffer + 12.5 mM $MgCl_2$ + 10 mM DTT at pH 8.0, 25 °C. Concentrations of red (R) and green (G) RNA fuels tested are indicated in the figure. Fluorescence microscopy images scale bar, 2.5 µm.*

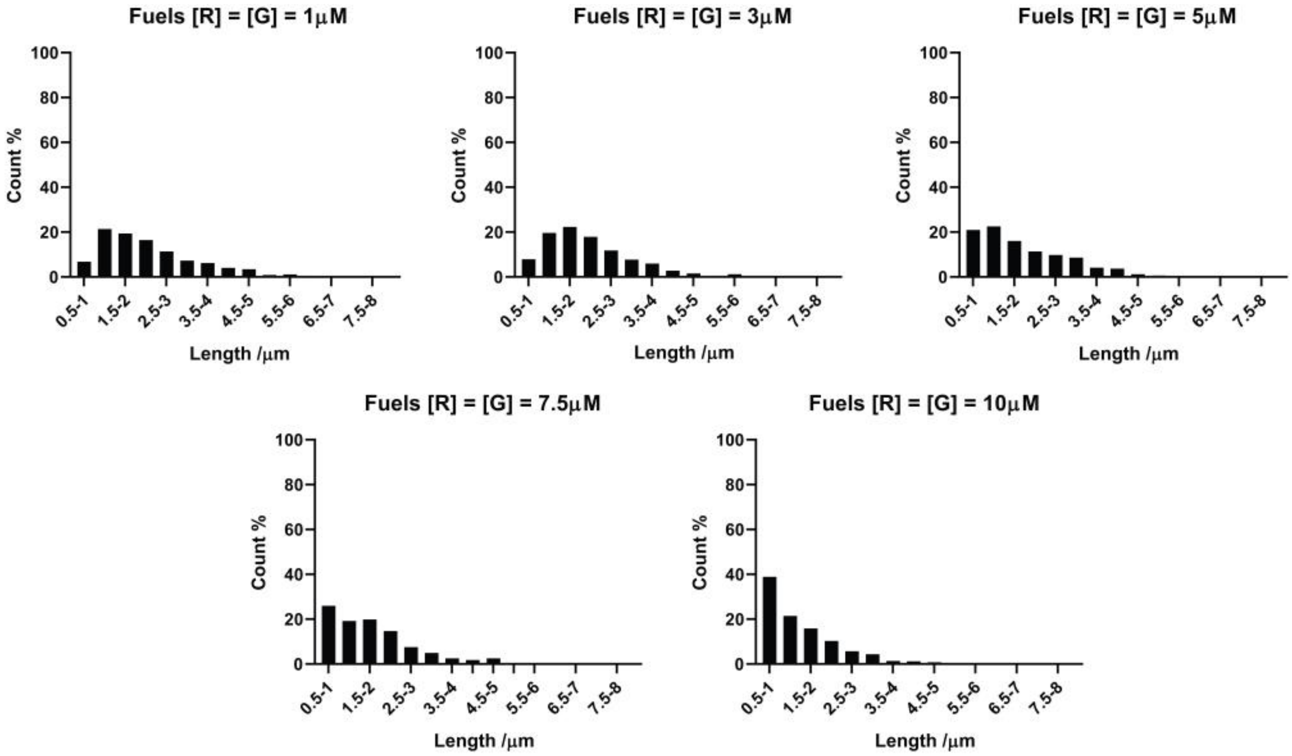

Figure S5. *Statistical analysis of DNA-based polymers count (%) vs. average length obtained at 6h from the confocal microscopy images of RNA-triggered reorganization at different RNA fuels concentrations shown in Figures 2d, S2. See legend of Figure 2 for experimental details. Each bar in the histogram plot shows the percentage number of DNA structures observed over a 0.5 μm range of polymer length.*

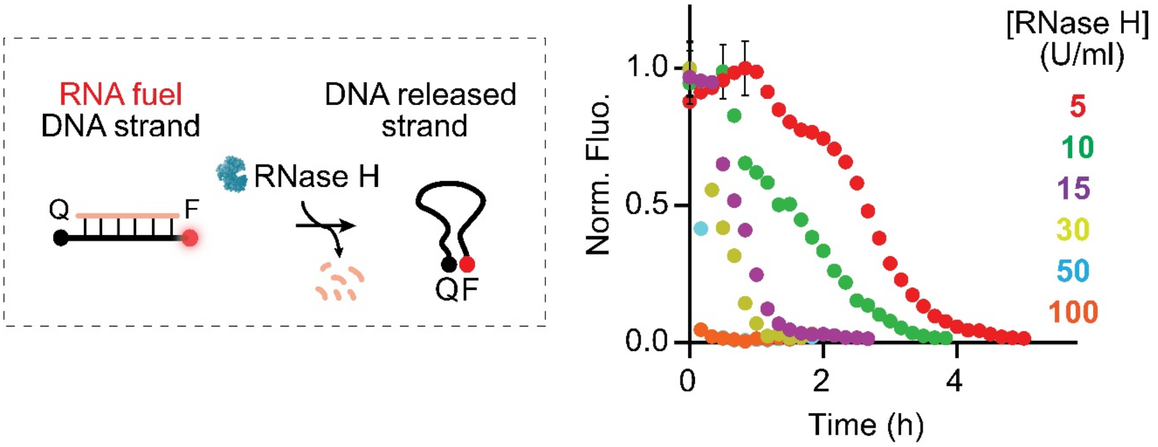

Figure S6. *Kinetic modulation of fuel degradation at different RNase H concentrations. Kinetic traces showing the degradation of an RNA strand bound to a fluorescent-labeled DNA complementary strand. The experiments were performed in 1 × TAE buffer + 12.5 mM $MgCl_2$ + 10 mM DTT, pH 8.0, 25 °C. [RNA fuel] = 3 μM and [fluorescent labeled DNA strand] = 250 nM. Concentrations of RNase H tested are indicated in the figure. For a matter of clarity, the error bars in the normalized values have been depicted for only one point on each kinetic trace and represent the maximum value of standard deviation.*

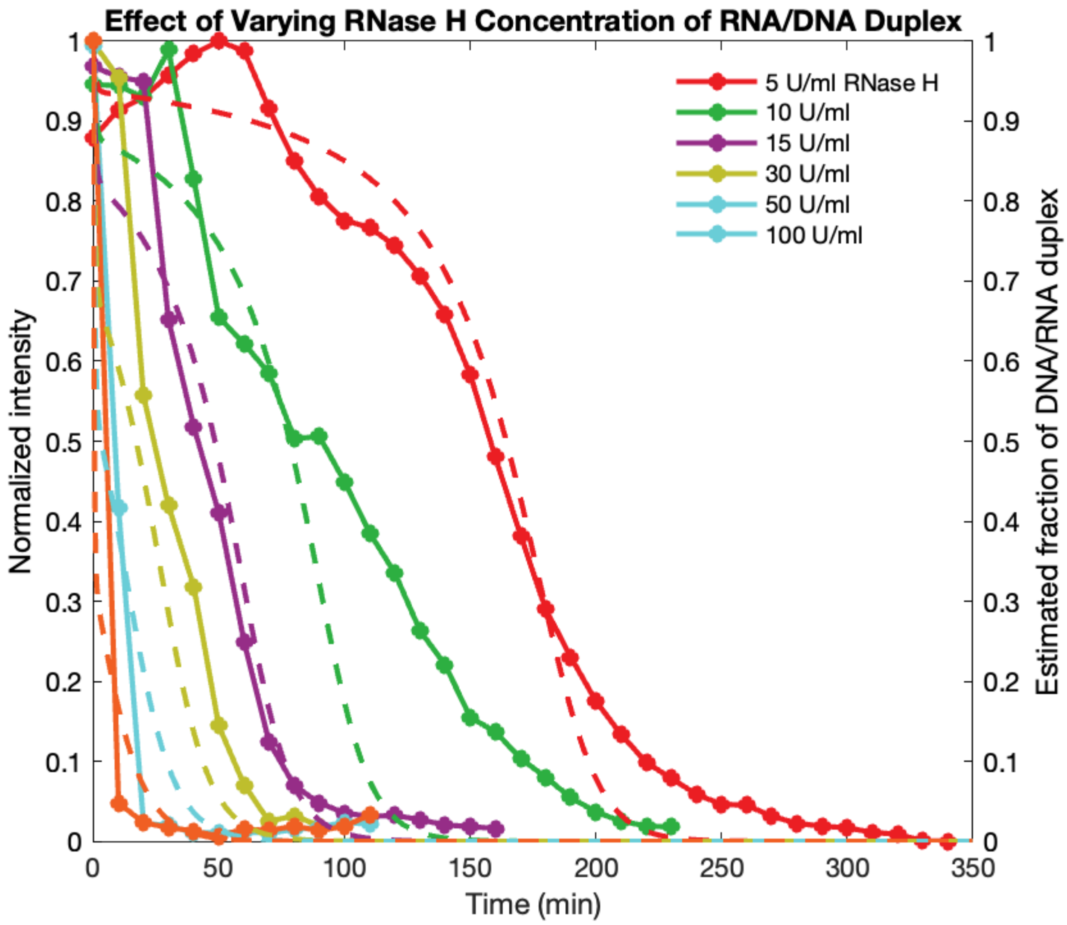

Figure S7. *Experimental kinetic data and computational prediction of DNA/RNA duplex fraction. The computational model (1)-(3) was fitted to experimental data. Dashed lines show the solutions of the ODE model adopting fitted parameters in Table S1.*

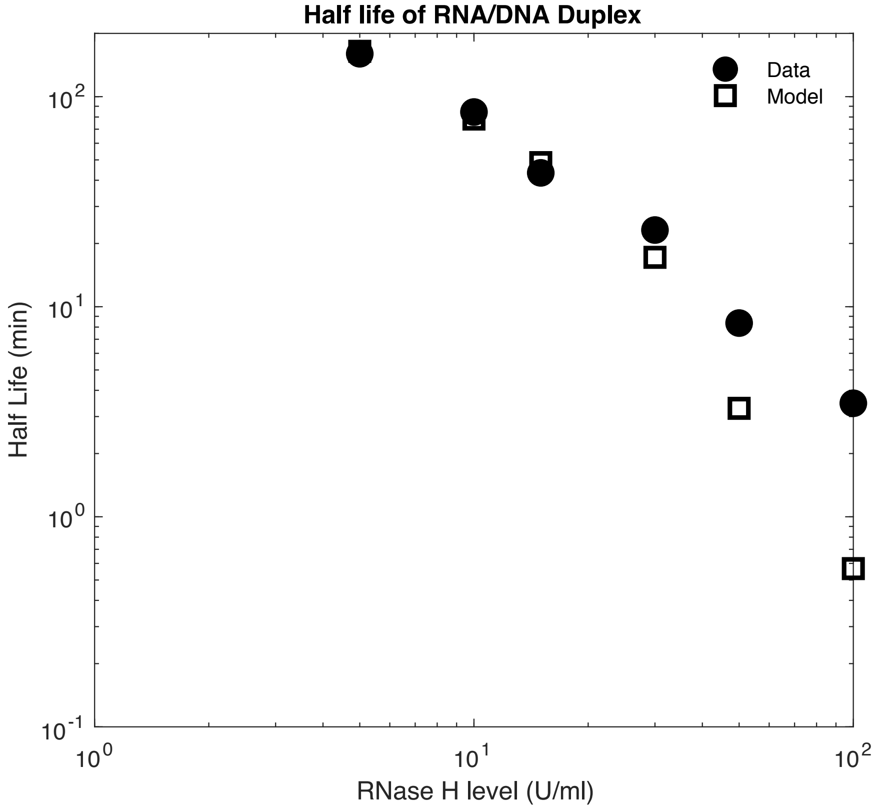

Figure S8. *Comparison between the measured and predicted half life of RNA/DNA duplex: The predicted half-life was computed from the trajectories in Fig. S7, generated by integrating model (1)-(3) adopting the fitted parameters in Table S1.*

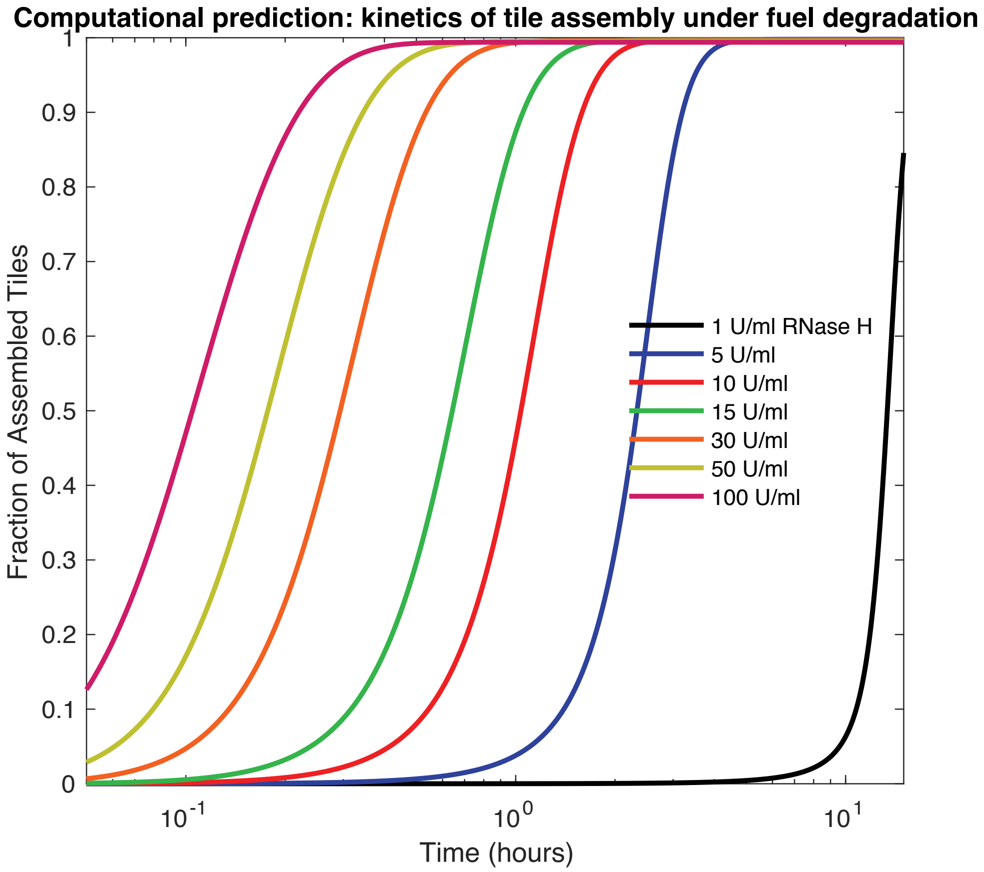

Figure S9. *Predicted tile assembly kinetics. The unfitted computational model (4)-(7) was integrated using MATLAB to generate predicted kinetic data for tile assembly. We show the predicted fraction of tiles that are assembled into nucleated lattices or nanotubes given different values of RNase H supplied to the sample.*

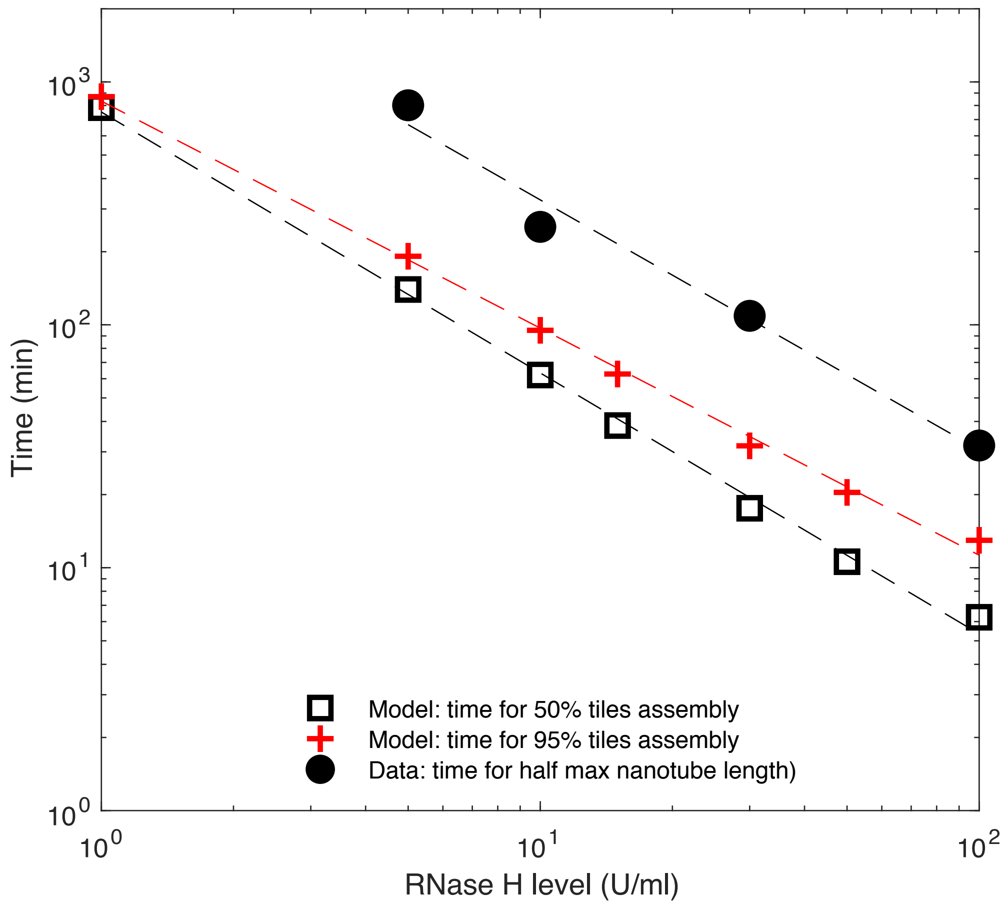

Figure S10. *Comparison between the time required for reaching half the maximal nanotube mean length (data) and the estimated time required to assemble 50% and 95% of the tiles (model). The unfitted computational model (4)-(7) was integrated using MATLAB to generate the predicted times (hybridization and RNase H parameters from Table S1, and tile assembly parameters from* Zhang *et al.* [2] *Dashed lines are an exponential (linear) fit in a log scale. The fitted slopes for data and predicted 50% tile assembly times are remarkably similar, suggesting that the model captures the relative reduction of assembly speed given an increase in RNase H. The difference between the observed timescale for nanotube growth and the predicted timescale of tile assembly can be explained by noting that the model does not capture nanotube joining, a prominent driver of nanotube growth*[1] *that proceeds more slowly than tile assembly into small lattices not visible via fluorescence microscopy. Further, our model does not capture phenomena of incomplete RNA degradation products that contribute to accumulation of inactive tiles (waste) and may slow down assembly.*[10]

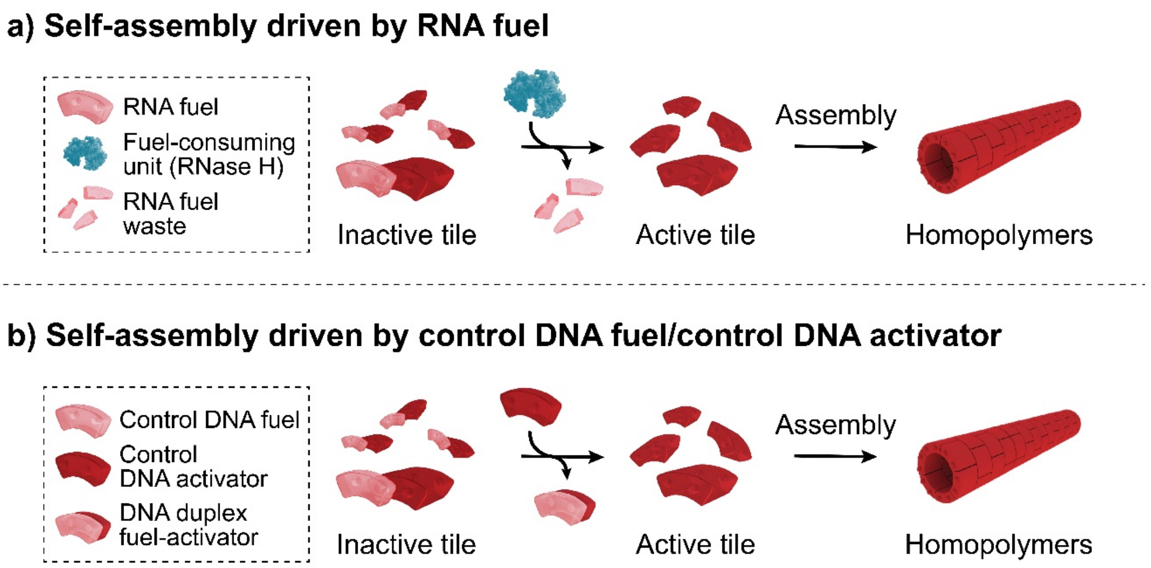

Figure S11. *General scheme of DNA tiles activation and self-assembly of DNA-based polymers driven by RNA fuels and enzymatic RNA degradation (a) or by using a control DNA fuel and control DNA activator (b).*[3,4]

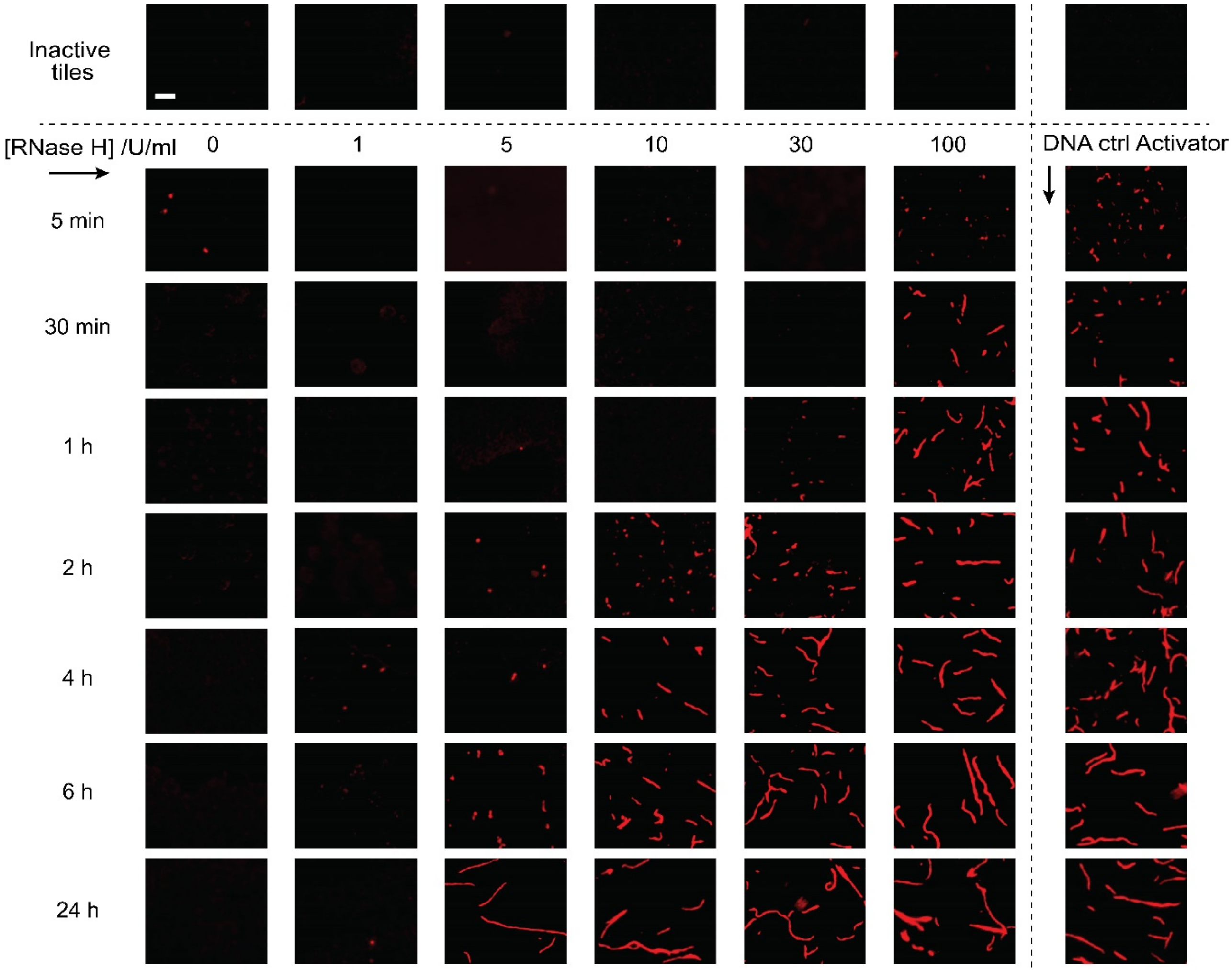

Figure S12. *Kinetic modulation of the DNA tiles assembly into homopolymers. The kinetic of the self-assembly of the R inactive tiles can be modulated by varying the concentration of RNase H in solution. The experiments were performed in presence of 0.25 μM of R homopolymers inactivated by 3 μM of RNA fuel (R) or 3 μM of control DNA Inhibitor (R)* [3,4]*. DNA homopolymers re-assembly was carried out in 1 × TAE buffer + 12.5 mM $MgCl_2$ at pH 8.0, 25 °C with different concentrations of RNase H (reported in the figure) or with 6 μM of control DNA Activator (R). Fluorescence images scale bar, 2.5 μm.*

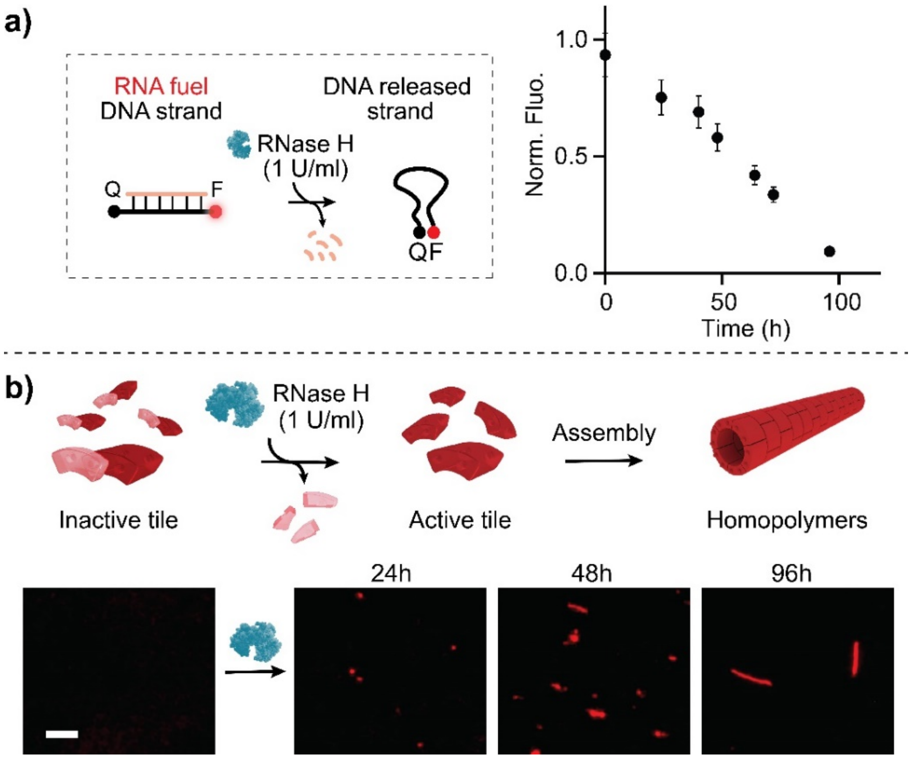

Figure S13. *(a) Kinetic of RNA fuel degradation at low level of RNase-H. Kinetic trace showing the degradation of an RNA strand bound to a fluorescent-labeled DNA complementary strand in the presence of a low concentration of RNase-H (1U/ml). The experiments were performed in 1 × TAE buffer + 12.5 mM $MgCl_2$ + 10 mM DTT, pH 8.0, 25 °C. [RNA fuel] = 3 μM and [fluorescent labeled DNA strand] = 250 nM. After 48h, 5 mM DTT was added into the solution to preserve the enzymatic activity. Error bars represent standard deviation based on triplicate measurements. (b) Kinetic of the formation of DNA-based polymers at low level of RNase-H. Fluorescence confocal images showing the self-assembly of the red (R) inactive tiles at low concentration of RNase-H (1U/ml). The experiment was performed in presence of 0.25 μM of red (R) homopolymers inactivated by 3 μM of RNA fuel (R). DNA homopolymers re-assembly was carried out in 1 × TAE buffer + 12.5 mM $MgCl_2$ + 10 mM DTT at pH 8.0, 25 °C. After 48h 5 mM DTT was added into the solution. Fluorescence images scale bar, 2.5 μm. Error bars represent standard deviation based on triplicate measurements.*

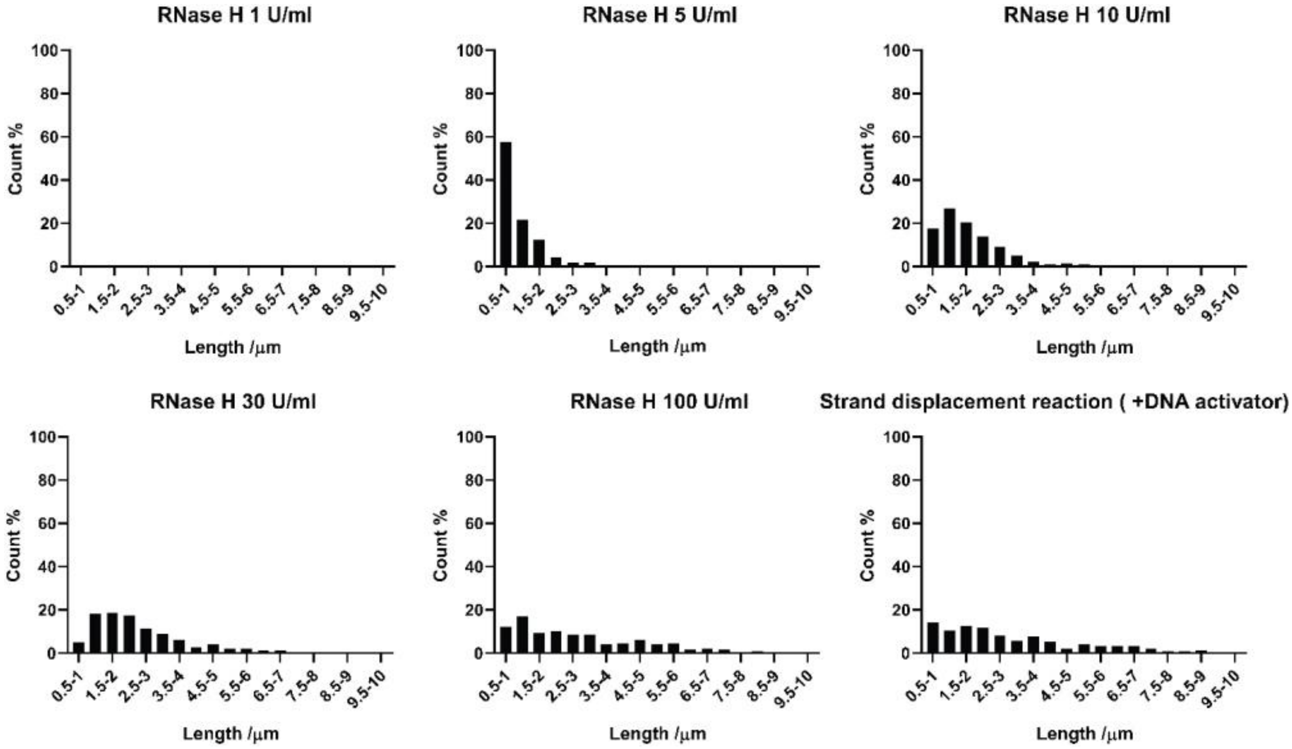

Figure S14. *Statistical analysis of DNA-based polymers count (%) vs. average length obtained at 6h from the confocal microscopy images of DNA polymer reorganization at different RNase H concentration shown in Figure 3c. See legend of Figure 3 for experimental details. Each bar in the histogram plot shows the percentage number of DNA structures observed over a 0.5 μm range of polymer length.*

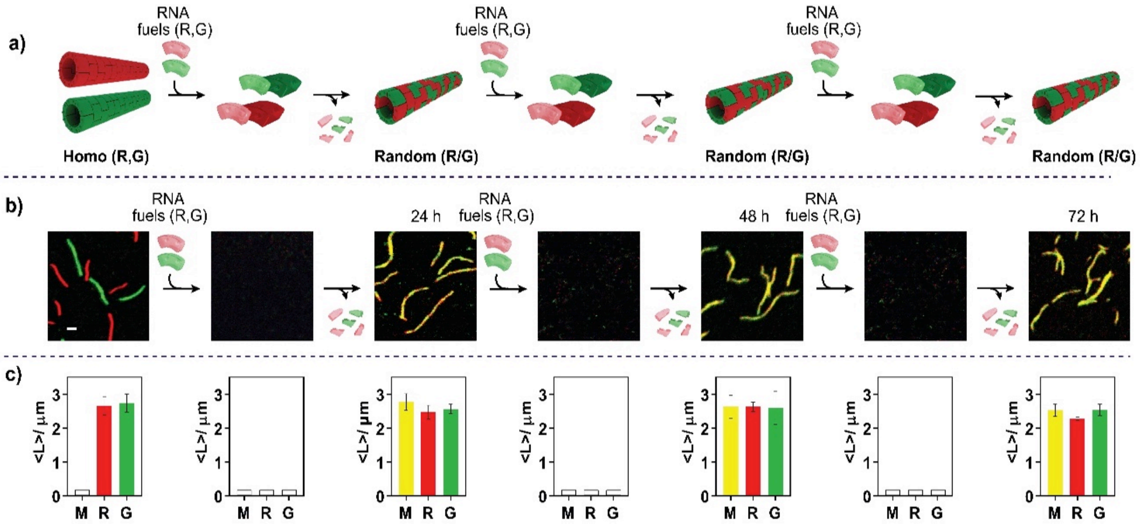

Figure S15. *Reversible reorganization cycles from R, G homopolymers to a R/G random co-polymer. Confocal images showing the reversible reorganization with successive addition of red and green RNA fuels. The experiments were performed in presence of 0.15 µM of both R,G homopolymers, 30 U/ml RNase H, 10 mM DTT in 1 × TAE buffer + 12.5 mM $MgCl_2$ at pH 8.0, 25 °C. Each cycle required the addition of 0.3 µM of both red and green RNA fuels. Confocal images scale bar, 2.5 µm.*

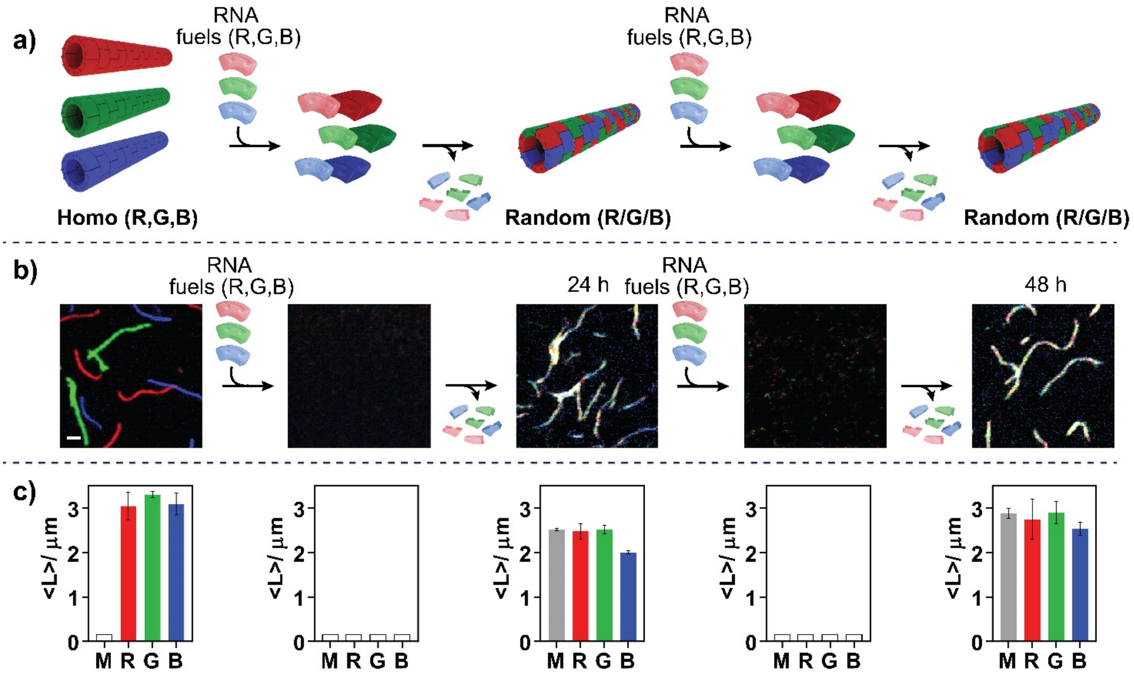

Figure S16. *Reversible reorganization cycles from R, G, B homopolymers to a R/G/B random co-polymer. Confocal images showing the reconfiguration following the successive addition of R,G,B RNA fuels in the presence of the three homopolymers. The experiments were performed in presence of 0.15 µM of homopolymers, 30 U/ml RNase H, 10 mM DTT in 1 × TAE buffer + 12.5 mM $MgCl_2$ at pH 8.0, 25 °C. Each cycle required the addition of 0.3 µM of R,G,B RNA fuels (for each cycle). Confocal images scale bar, 2.5 µm.*

Supplemental References

[1] P. W. Rothemund, A. Ekani-Nkodo, N. Papadakis, A. Kumar, D. K. Fygenson, E. Winfree. *J. Am. Chem. Soc*. **2004**, *126*, 16344–16352.

[2] D. Y. Zhang, R. F. Hariadi, H. M. Choi, E. Winfree. *Nat. Commun*. **2013**, *4*, 1965.

[3] L. N. Green, A. Amodio, H. K. K. Subramanian, F. Ricci, E. Franco. *Nano Lett*. **2017**, *17*, 7283–7288.

[4] L. N.Green, H. K. K. Subramanian, V. Mardanlou, J. Kim, R. F. Hariadi, E. Franco. *Nat. Chem*. **2019**, *11*, 510–520.

[5] S. Gentile, E. Del Grosso, L. J. Prins, F. Ricci. *Angew. Chem. Int. Ed*. **2021**, *60*, 12911–12917.

[6] S. Bolte, F. P. Cordelières. *J. Microsc*. **2006**, *224*, 213–232.

[7] S. V. Costes, D. Daelemans, E. H. Cho, Z. Dobbin, G. Pavlakis, S. Lockett. *Biophys. J.* **2004**, *86*, 3993-4003.

[8] J. Kim, E. Winfree. *Mol Syst Biol* **2011**, *7*, 465.

[9] E. Franco, E. Friedrichs, J. Kim, R. Jungmann, R. Murray, E. Winfree, F. C. Simmel. *Proc Natl Acad Sci USA.* **2011**, *108*, 784-793.

[10] S. Agarwal, M. A. Klocke, P. E. Pungchai, E. Franco. *Nat Commun* **2021**, *12*, 3557.
